# A responder-informed gut microbial consortium enhances anti-PD-1 efficacy in a mouse cancer model

**DOI:** 10.20517/mrr.2025.117

**Published:** 2026-02-09

**Authors:** Uk Jin Jeong, Mohammed Ali, Yun Jee Park, Jin Sun You, Sang Sun Yoon

**Affiliations:** ^1^Department of Microbiology and Immunology, Yonsei University College of Medicine, Seoul 03722, Republic of Korea.; ^2^Brain Korea 21 Project for Medical Sciences, Yonsei University College of Medicine, Seoul 03722, Republic of Korea.; ^3^Section of Hematology, Yale Cancer Center, Yale University School of Medicine, New Haven, CT 06511, USA.; ^4^Institute for Immunology and Immunological Diseases, Yonsei University College of Medicine, Seoul 03722, Republic of Korea.; ^5^Severance Biomedical Science Institute, Yonsei University College of Medicine, Seoul 03722, Republic of Korea.; ^6^BioMe Inc., Seoul 02455, Republic of Korea.

**Keywords:** Gut microbiota, cancer immunotherapy, immune checkpoint inhibitors, anti-PD-1, tumor microenvironment, host-microbiome interactions

## Abstract

**Aim:** Immune checkpoint inhibitors (ICIs), particularly anti-programmed cell death protein 1 (PD-1) therapy, have improved cancer treatment outcomes, yet durable benefit is achieved in only a subset of patients. Growing evidence implicates the gut microbiome as a modulator of ICI responsiveness, but defined and experimentally validated microbial strategies remain limited. This study aimed to identify responder-associated gut microbes and to evaluate a defined bacterial consortium for enhancing PD-1 blockade efficacy.

**Methods:** Publicly available shotgun metagenomic datasets from anti-PD-1-treated cancer patients were re-analyzed to compare gut microbiome profiles between responders and non-responders. Bacterial taxa reproducibly enriched in responders were selected based on consistency across analytical criteria and cultivability and assembled into a four-strain consortium (UJ-04). The immune-adjuvant potential of UJ-04, alone or combined with anti-PD-1 therapy, was evaluated in a B16-F10 melanoma mouse model, with tumor growth and immune responses assessed by flow cytometry.

**Results:** Metagenomic re-analysis identified four commensal bacterial taxa consistently enriched in responder patients, forming the defined UJ-04 consortium. While UJ-04 alone showed minimal antitumor activity, combination treatment with anti-PD-1 significantly enhanced tumor growth inhibition compared with anti-PD-1 monotherapy. This effect was accompanied by increased intratumoral CD8^+^ T cells and natural killer cells, with concordant immune trends in peripheral compartments.

**Conclusion:** A responder-informed, defined microbial consortium functionally translates clinical microbiome associations into *in vivo* validation and enhances PD-1 blockade efficacy by modulating host antitumor immunity. These findings support defined bacterial consortia as microbiome-based immunomodulatory adjuncts for immunotherapy.

## INTRODUCTION

Immune checkpoint inhibitors (ICIs), particularly agents targeting the programmed cell death protein 1 (PD-1)/programmed cell death ligand 1 (PD-L1) axis, have transformed the management of solid tumors by releasing inhibitory pathways that restrain antitumor T cell activity^[1]^. [and, in selected settings, cytotoxic T-lymphocyte-associated protein 4 (CTLA-4)] have produced some of the most striking clinical successes, yielding durable remissions in a subset of patients and establishing new standards of care across multiple tumor types^[2-4]^. These results demonstrate that, when inhibitory signals are appropriately blocked and effector cells are effectively recruited, endogenous immunity can exert potent and sustained antitumor effects^[5,6]^.

Mechanistically, chronic antigen exposure within the tumor microenvironment (TME) drives persistent T-cell activation and interferon-γ production, leading to adaptive upregulation of PD-L1 on tumor and stromal cells and reinforcement of inhibitory PD-1 signaling^[7]^. Antibody-mediated blockade of PD-1 or PD-L1 disrupts this inhibitory axis, restores T-cell receptor signaling and effector function, and enables immune-mediated tumor cell killing^[8]^.

Despite these breakthroughs, objective response rates for many malignancies remain low, and resistance (both intrinsic and acquired) is widespread^[6,9,10]^. In routine practice, only a subset of patients achieves meaningful and durable benefit from PD-1/PD-L1 blockade, while the majority experience primary resistance or eventually develop disease progression^[11,12]^. Although immune checkpoint therapy consistently improves survival compared with conventional chemotherapy, long-term follow-up demonstrates continued loss of benefit over time, indicating that response durability remains insufficient for many patients^[13]^. Even intensified combination strategies have not fully overcome these limitations, underscoring that blockade of the PD-1/PD-L1 axis alone is insufficient to elicit sustained antitumor immunity in most cases^[14]^.

Contributing factors include insufficient infiltration of effector T cells into tumors^[15,16]^, impaired antigen presentation and interferon signaling^[17,18]^, and the accumulation of immunosuppressive myeloid populations within the TME^[19]^. These limitations motivate adjunct strategies capable of improving cytotoxic lymphocyte trafficking and function within tumors, including approaches that modulate host-environmental factors such as the gut microbiota^[20,21]^.

Converging clinical and preclinical evidence implicates the gut microbiota as a modulator of response to PD-1 blockade. Cohort studies have associated responder status with distinct microbial signatures, frequently enriched for commensals linked to improved antitumor immunity, while transfer or enrichment of defined taxa or consortia has been shown to augment T-cell-mediated responses *in vivo*^[22-29]^. Mechanistically, microbiome influences on antigen presentation^[30]^, interferon signaling, and chemokine networks have been implicated in improved recruitment and licensing of tumor-infiltrating lymphocytes (TILs)^[24,25,29]^. Yet, despite these advances, many studies examine individual components of this process in isolation, and relatively few have integrated clinical metagenomic re-analysis with functional *in vivo* validation. To address this gap, we followed a two-step approach. First, we re-analyzed publicly available metagenomic datasets^[23]^ from patients treated with anti-PD-1 to nominate taxa enriched in responders versus non-responders according to response evaluation criteria in solid tumors (RECIST) v1.1^[31]^. Second, informed by these patterns, we assembled a defined microbial consortium (UJ-04) and tested its ability to potentiate anti-PD-1 therapy in the B16-F10 melanoma model.

Importantly, the objective of the *in vivo* experiments was not to establish tumor-type-specific therapeutic efficacy, but rather to address a broader immunological question: whether a responder-associated microbial consortium can function as an immunomodulatory adjunct that enhances the efficacy of PD-1 blockade. In this context, we employed the B16-F10 melanoma model as a well-characterized and immunologically stringent system to evaluate whether a responder-biased microbial consortium (UJ-04) can function as an immunomodulatory adjunct that potentiates PD-1 blockade *in vivo*. By integrating tumor growth assessment with endpoint immune profiling of T-cell and natural killer (NK)-cell compartments, this study directly links clinical metagenomic re-analysis to functional *in vivo* validation.

## METHODS

### Comparative metagenome analysis

We re-analyzed a previously published shotgun metagenomic dataset from patients with advanced non-small-cell lung cancer (NSCLC) treated with PD-1 blockade^[23]^. The original cohort comprised 338 patients who received anti-PD-1 therapy. Clinical response categories were defined according to RECIST criteria^[31]^ in the original study. Patients with complete response (CR), partial response (PR), or stable disease (SD) were classified as responders, whereas patients with progressive disease (PD) were classified as non-responders. Patients with recent antibiotic exposure, as reported in the original metadata, were excluded from all analyses. After this exclusion, 269 patients (155 responders and 114 non-responders) were retained for downstream microbiome analyses. Baseline clinical and demographic characteristics of the responder and non-responder groups are summarized in Supplementary Table 1.

Species-level taxonomic profiles derived from shotgun metagenomic sequencing were obtained from the publicly available, pre-processed taxonomic abundance tables provided by the original study, rather than from raw sequencing reads. All downstream analyses were performed in R (version 4.5.2).

Sample identifiers from the taxonomic abundance table and the clinical metadata were harmonized by converting sample IDs to a consistent format and matching row names between datasets. Only samples present in both datasets were retained for analysis. Taxonomic profiling and annotation were performed in the original publication using the authors’ established bioinformatics pipeline and reference databases, as described previously^[23]^, and these annotations were used directly in the present study.

To focus specifically on bacterial community composition, non-bacterial features were removed by retaining only taxa annotated as belonging to the bacterial kingdom. All features annotated as eukaryotic, viral, or archaeal were excluded. After removal of non-bacterial taxa, a total of 605 bacterial species remained. No additional hard filtering thresholds based on minimum abundance or prevalence were applied at this stage, to retain the full set of bacterial species for exploratory screening.

Relative abundance values were normalized on a per-sample basis. For initial screening and prioritization of response-associated taxa, species-level relative abundances were additionally transformed using min-max normalization across samples to capture relative directionality and dynamic range. For each species, the difference between the mean normalized abundance in responders and non-responders was calculated and used as a ranking metric in the initial abundance-based screening step.

Prevalence was defined as the proportion of samples in which a given species exhibited non-zero abundance within each response group. Species were evaluated jointly based on relative abundance differences and prevalence patterns to identify taxa consistently associated with treatment response. Differential abundance analysis was performed using Linear Discriminant Analysis Effect Size (LEfSe)^ [32]^, with response status (responder *vs.* non-responder) specified as the class variable. LEfSe was applied using a Kruskal-Wallis α-level of 0.05 and a logarithmic Linear Discriminant Analysis (LDA) score threshold of 2.0, and taxa meeting these criteria at the genus and species levels were retained for downstream analyses.

### Overall survival analysis

Overall survival (OS) was analyzed for bacterial species that showed a positive association with treatment response in the preceding microbiome analyses. For each selected species, baseline species-level relative abundance was extracted for all eligible patients. Patients were stratified into “high” and “low” abundance groups using the cohort median as the cutoff.

OS was compared between the high- and low-abundance groups using Kaplan-Meier survival curves with log-rank testing. In addition, exploratory Cox proportional hazards models were fitted to estimate hazard ratios, both unadjusted and adjusted for available baseline covariates, including age, sex, and Eastern Cooperative Oncology Group (ECOG) performance status.

Because other clinically relevant factors known to influence survival in advanced NSCLC - such as smoking history, tumor histology, disease stage, and treatment line or regimen - were not available at the individual-patient level in the public dataset, these variables could not be included in multivariable models. Accordingly, OS analyses were performed as exploratory and hypothesis-generating assessments rather than definitive tests of independent microbiome effects.

### Growth curve

Growth characteristics were assessed to evaluate the *in vitro* cultivability of candidate strains identified through metagenomic screening. Each strain was activated in seed culture under anaerobic conditions (5% CO_2_, 5% H_2_, and 90% N_2_) and subsequently inoculated into fresh Gifu Anaerobic Medium (GAM, KisanBio, South Korea) and incubated in an anaerobic chamber at 37 °C. Optical density at 600 nm (OD_600_) was measured every 30 min for 24 h to generate growth curves for each strain. 

### Preparation of UJ-04 consortium

The defined microbial consortium UJ-04 consists of four commensal anaerobic bacterial species: *Anaerostipes hadrus (A. hadrus), Roseburia intestinalis (R. intestinalis)*, *Ruminococcus faecis (R. faecis)* and *Eubacterium rectale (E. rectale). *Details of the bacterial strains included in the UJ-04 consortium, including strain source and culture conditions, are provided in Supplementary Table 2.* R. intestinalis* and *E. rectale* were obtained from the Korean Collection for Type Cultures (KCTC, Jeongeup, South Korea). Both *R. faecis* and *A. hadrus*, which were isolated from healthy donors, were obtained from BioMe Inc.’s own human microbiome collection, YSFlora®^[33]^. All anaerobic media were pre-reduced for 48 h prior to use, and bacterial cultures were subsequently incubated at 37 °C under anaerobic conditions (5% CO_2_, 5% H_2_, and 90% N₂). Gifu Anaerobic Medium (KisanBio, South Korea) was used to cultivate UJ-04 strains. After cultivation, UJ-04 bacterial pellets were obtained by centrifugation at 4,750 revolutions per minute (rpm) for 20 min, followed by removal of the supernatant. The cells were then resuspended and rinsed once with sterile 1× phosphate-buffered saline (PBS; Sigma, USA). After washing, four strains were combined, each adjusted to a concentration of 1 × 10^8^ colony forming unit (CFU), and suspended in PBS containing 12.5% glycerol (Supelco, USA). The prepared mixture was aliquoted and stored at -80 °C until further use. Mice received UJ-04 (4 × 10^8^ CFU) via oral gavage once daily for 5 consecutive days.

### Animal husbandry

Seven-week-old female C57BL/6 mice were obtained from Orient Bio (Sungnam, Republic of Korea) and allowed to acclimatize for 1 week prior to experimentation. Animals were housed under controlled conditions (temperature, 24 ± 0.5 °C; relative humidity, 55%-65%) with an automatic 12-h light/dark cycle. Food and water were provided ad libitum. All animal procedures were conducted in accordance with the institutional guidelines of the Department of Animal Resources at the Yonsei Avison Biomedical Research Center (ABMRC). The experimental protocol was reviewed and approved by the Institutional Animal Care and Use Committee of Yonsei University College of Medicine (approval number: 2025-0275).

### Animal experimental design and grouping

All *in vivo* experiments were performed using 7-week-old female C57BL/6 mice. Tumor growth experiments were initiated with 8-10 mice per treatment group, followed by antibiotic-mediated gut microbiota depletion and subcutaneous implantation of B16-F10 melanoma cells. When tumor volumes reached a comparable size across groups (approximately 30-50 mm^3^), mice were randomly assigned to experimental groups to minimize allocation bias. To evaluate the effect of the UJ-04 consortium on PD-1 blockade efficacy, mice were assigned to four treatment groups: (i) isotype control antibody plus PBS; (ii) isotype control antibody plus UJ-04; (iii) anti-PD-1 antibody plus PBS; and (iv) anti-PD-1 antibody plus UJ-04.

During the course of the experiment, mice that met predefined exclusion criteria, including tumor implantation failure, abnormal tumor growth, or humane endpoint criteria, were excluded from the analysis. These criteria were applied uniformly across all treatment groups. The final analysis included 4-7 mice per group, and the exact number of animals used in each experiment and independent repeat is specified in the corresponding figure legends. Independent replicate experiments were conducted using the same experimental design to ensure reproducibility. Tumor growth was monitored longitudinally at identical time points across groups, and tumor volumes were calculated using a consistent measurement method for all treatment groups.

### Antibiotics treatment

To deplete the gut microbiota, starting 7 days before tumor implantation, mice received drinking water containing ampicillin sodium salt (1 g/L; Sigma-Aldrich, USA), streptomycin sulfate (3 g/L; Duchefa, The Netherlands), and colistin sulfate (1 g/L; KisanBio, South Korea) prepared in autoclaved tap water. To mask bitterness and promote intake, sucrose (1% w/v; Junsei, Japan) was added. Antibiotic drinking water was replaced with freshly prepared solution daily and provided ad libitum.

### Cell culture

The Korean Cell Line Bank (KCLB, South Korea) provided the B16-F10 melanoma cells. Cells were maintained at 37 °C in a humidified incubator with 5% CO_2_ and cultured in high-glucose Dulbecco’s Modified Eagle Medium (DMEM; Welgene, South Korea) supplemented with 10% fetal bovine serum (FBS; Gibco, USA) and 1% penicillin-streptomycin (P/S, Welgene, South Korea). Cells were passaged at 70%-80% confluence and, prior to *in vivo* use, were expanded for ≥ 3 passages**; **log-phase cells were used for all experiments. Routine mycoplasma testing was performed and only mycoplasma-negative cultures were used. 

For tumor inoculation, cells were washed once with 1× Dulbecco’s phosphate-buffered saline (DPBS), detached with trypsin-ethylenediaminetetraacetic acid (EDTA), pelleted (1,000 rpm, 3 min), and washed once with DPBS. Viable cells were counted and resuspended in DPBS at 3 × 10^6^ cells/mL**.**

### Tumor-bearing mouse model

Before implantation, mice were anesthetized by intraperitoneal injection of ketamine/xylazine in PBS (ketamine:xylazine:PBS = 2.5:0.4:7.2). Each mouse was subcutaneously injected with 100 µL of the suspension **(**3 × 10^5^ cells**)** (right flank). Starting 7 days after tumor cell implantation, when the tumor volume reached approximately 30-50 mm^3^, mice were intraperitoneally administered either anti-PD-1 antibody (BioXcell, USA) or an immunoglobulin G subclass 2 (IgG2) isotype control (BioXcell, USA) at a dose of 100 µg per mouse. The injections were given once every three days for a total of five treatments. Tumor dimensions were recorded at the same interval, and the volume (V) was determined using V = (L × W^2^) / 2, where L represents the longest axis and W denotes the perpendicular shorter axis. Mice were euthanized humanely if any of the following endpoints were reached: tumor diameter exceeding 2 cm, tumor volume surpassing 2,000 mm^3^, or visible signs of ulceration, hemorrhage, or necrosis.

### Flow cytometry analysis

Following euthanasia, tumors and spleens were aseptically harvested from tumor-bearing mice at the indicated experimental endpoints. Excised tumors were weighed and processed into single-cell suspensions for flow cytometric analysis. Briefly, tumor tissues were carefully trimmed to remove connective tissue and capsules, finely minced, and enzymatically digested in high-glucose DMEM containing collagenase IV and DNase I supplemented with FBS at 37 °C with gentle agitation. After digestion, cell suspensions were filtered through 70 µm cell strainers to remove debris, washed with cold Magnetic-Activated Cell Sorting (MACS) buffer to terminate enzymatic activity, and pelleted by centrifugation.

Spleens were mechanically dissociated through 70 µm cell strainers into cold MACS buffer, followed by red blood cell lysis according to the manufacturer’s instructions. Cells were subsequently washed and resuspended for downstream assays. Where indicated, splenocytes were plated in 96-well round-bottom plates for *ex vivo* stimulation.

The MACS buffer consisted of PBS supplemented with 0.5% FBS and 2 mM EDTA and was maintained on ice throughout processing. For functional cytokine analysis, cells were stimulated for 4 h at 37 °C in a humidified 5% CO_2_ incubator using the eBioscience**^TM^** Cell Stimulation Cocktail (500×; Invitrogen), which contains phorbol 12-myristate 13-acetate (PMA) and ionomycin to induce cellular activation and cytokine production. No protein transport inhibitors were included during stimulation.

Following stimulation, cells were stained for surface markers for 30 min at 4 °C in the dark. Cells were then fixed and permeabilized according to the manufacturer’s protocol, followed by intracellular staining. A fixable live/dead viability dye was included where applicable to exclude nonviable cells.

Data acquisition was performed on a BD FACSCelesta**^TM^** flow cytometer (BD Biosciences), and analyses were conducted using FlowJo software (version 10.10.0). Cells were gated sequentially on singlets, live cells, and CD45^+^ leukocytes, followed by lineage-specific markers to identify immune cell subsets of interest. Analyses focused on immune populations that showed reproducible and biologically meaningful differences between experimental groups. Immune cell frequencies were calculated as percentages of parent populations, and, where applicable, absolute cell numbers were normalized to tumor weight.

### Serum preparation

Blood was collected from tumor-bearing C57BL/6 mice via the abdominal aorta immediately after sacrifice. The samples were left at room temperature for approximately 30 min to allow clotting, and then centrifuged at 2,000 rpm for 10 min. The supernatant serum was aliquoted and stored at -80 °C until use.

### Cytometric bead array

Serum cytokine and chemokine profiling was performed to assess systemic immune responses associated with treatment using a multiplex cytometric bead array (CBA, LEGENDplex™ Mouse Cytokine Release Syndrome Panel (13-plex) w/ VbP, BioLegend, CA, USA). The panel detected: interferon gamma (IFN-γ), interferon alpha (IFN-α), tumor necrosis factor alpha (TNF-α), interleukin 4 (IL-4), interleukin 6 (IL-6), interleukin 10 (IL-10), granuocyte-macrophage colony-stimulating factor (GM-CSF), C-C motif chemokine ligand 2 (CCL2), C-C motif chemokine ligand 3 (CCL3), C-C motif chemokine ligand 4 (CCL4), C-X-C motif chemokine ligand 9 (CXCL9), and C-X-C motif chemokine ligand 10 (CXCL10). Before the assay, serum was thawed on ice and diluted 1:2 in assay buffer. For each well, 25 μL of Matrix A, 25 μL of diluted sample or standard, and 25 μL of mixed capture beads were combined in a V-bottom 96-well plate and incubated for 2 h at room temperature on a plate shaker (~ 800 rpm) in the dark. After a wash step, 25 μL of detection antibody mixture was added and incubated for 1 hr, followed by 25 μL of SA-PE for 30 min under the same conditions. Beads were washed again, resuspended in 150 μL of wash buffer, and acquired at low flow on a flow cytometer (FACSCelesta**^TM^**, BD) with at least ~ 300 events per analyte. Concentrations were calculated from standard curves using the LEGENDplex**^TM^** data analysis software with a 5-parameter logistic fit; the zero standard was treated as blank.

### Statistical analysis

Data are presented as mean ± standard error of the mean (SEM) unless otherwise indicated. Serum cytokine and chemokine concentrations measured by CBA are reported as median values. Statistical analyses were performed using GraphPad Prism (version 10.1.1; GraphPad Software Inc., La Jolla, CA, USA) and R (version 4.5.2; R Foundation for Statistical Computing, Vienna, Austria). All statistical tests were two-sided, and *P* < 0.05 was considered statistically significant.

For comparative metagenomic analyses, relative abundances were compared between responders and non-responders using the Mann-Whitney *U* test. LEfSe was performed using a Kruskal-Wallis α-level of 0.05 and a logarithmic LDA score threshold of 2.0 to identify taxa differentially associated with response.

OS in the clinical cohort was analyzed for microbial species showing positive associations with response. Patients were stratified into high- and low-abundance groups based on the median baseline relative abundance, and survival differences were assessed using Kaplan-Meier analysis with log-rank tests.

For *in vivo* tumor experiments, tumor growth over time was analyzed by two-way analysis of variance (ANOVA) with treatment group and time as factors, followed by Sidak’s post hoc test. Tumor weight at the experimental endpoint, flow cytometry readouts, and serum cytokine or chemokine levels measured by CBA were compared among groups using the Kruskal-Wallis test. Mouse survival was analyzed using the Kaplan-Meier method with log-rank tests.

## RESULTS

### Responder microbiomes are enriched for commensal Firmicutes taxa

**Figure 1:**
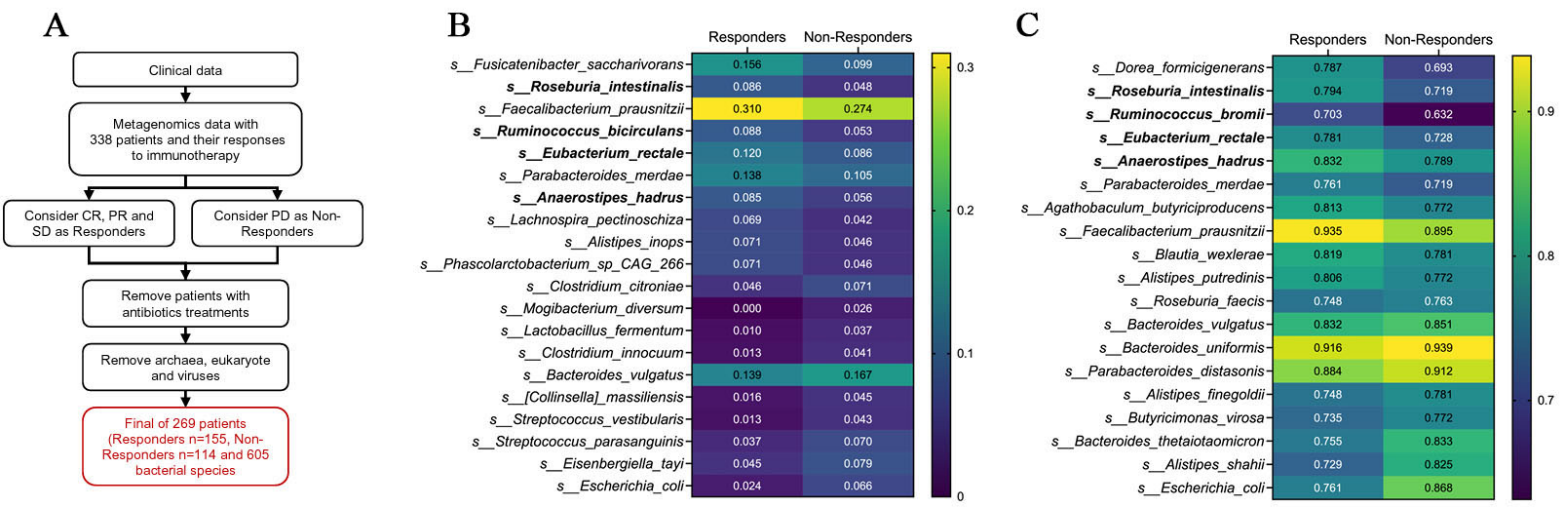
Abundance- and prevalence-based screening of response-associated bacterial species. (A) Schematic overview of the metagenomic re-analysis pipeline. Shotgun metagenomic data from patients with advanced NSCLC treated with anti-PD-1 therapy were re-analyzed. After exclusion of patients with recent antibiotic exposure and removal of non-bacterial features, 269 patients [responders (R), *n* = 155; non-responders (NR), *n* = 114] and 605 bacterial species were retained for downstream analysis; (B) Heatmap of mean relative abundance for species ranked by abundance mean difference (Δabundance = meanResponder - meanNon-responder) calculated from feature-wise min-max-scaled values. The top 10 responder-enriched and top 10 non-responder-enriched species are shown using the original relative abundance data; (C) Heatmap of prevalence for species ranked by prevalence difference (Δprevalence = prevResponder - prevNon-responder). Among species with prevalence ≥ 70% in responders, the top responder- and non-responder-enriched taxa are shown. CR: Complete response; PR: partial response; SD: stable disease; PD: progressive disease; NSCLC: non-small-cell lung cancer.

We re-analyzed a published shotgun metagenomic dataset from patients with advanced NSCLC treated with anti-PD-1 therapy. The original cohort included 338 patients. Based on best overall response metadata, patients with CR, PR, or SD were classified as responders, whereas patients with PD were classified as non-responders. After excluding patients with recent antibiotic exposure, 269 patients (155 responders and 114 non-responders) were retained for downstream microbiome analyses. From the taxonomic profiles, non-bacterial features, including eukaryotic taxa, viruses, and archaea, were removed, yielding a final dataset comprising 605 bacterial species [Figure 1A].

To nominate microbial taxa associated with response, we adopted a multi-step, multi-criteria prioritization framework rather than relying on a single statistical metric. As an initial screen, species-level relative abundances were compared between responders and non-responders using a min-max-scaled representation of the data to capture directionality and dynamic range across the cohort. This screening highlighted a set of responder-enriched taxa that were predominantly commensal Firmicutes, including *Fusicatenibacter saccharivorans*, *R. intestinalis*, *Faecalibacterium prausnitzii (F. prausnitzii)*, *Ruminococcus bicirculans*,* E. rectale*,* A. hadrus*, and *Lachnospira pectinoschiza*, whereas non-responder-enriched species included *Escherichia coli, Streptococcus *spp*.*,* Bacteroides vulgatus*, and other taxa commonly associated with dysbiosis [Figure 1B].

Because enrichment based on abundance alone does not capture cohort-level consistency, we next evaluated prevalence, defined as the proportion of samples in which each species was detected. When restricting the analysis to species present in at least 70% of responders, several of the same commensal Firmicutes taxa - including *R. intestinalis, A. hadrus, E. rectale, Ruminococcus bromii, *and* F. prausnitzii* - remained prominent, indicating that these taxa were not only enriched but also broadly distributed among responding patients. In contrast, multiple *Bacteroides *and* Escherichia* species showed higher prevalence in non-responders. Together, abundance- and prevalence-based screening steps yielded a focused set of candidate taxa consistently associated with response or non-response across complementary criteria [Figure 1C]. 

**Figure 2:**
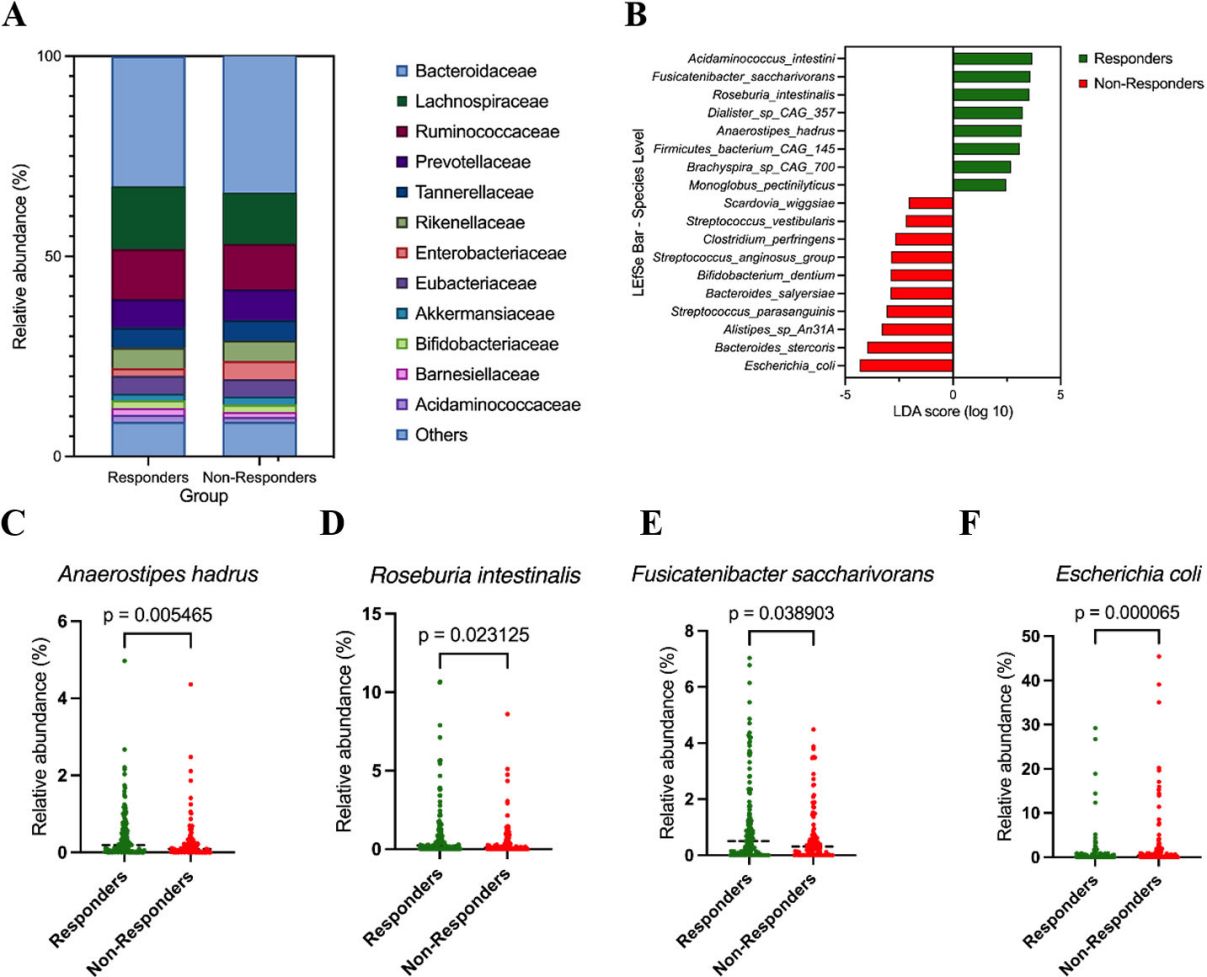
Taxonomic differences between responders and non-responders. (A) Family-level composition of the gut microbiota in responders (R, *n* = 155) and non-responders (NR, *n* = 114). Stacked bar plots show the mean relative abundance (%) of major bacterial families; minor families are grouped as “Others”; (B) LEfSe analysis at the species level comparing responders (R, *n* = 155) and non-responders (NR, *n* = 114); (C-F) Relative abundance of selected species in responders and non-responders. Panels show *Anaerostipes hadrus* (C), *Roseburia intestinalis* (D), *Fusicatenibacter saccharivorans* (E), and *Escherichia coli* (F). Relative abundances are expressed as percentages. Group comparisons were performed using two-sided Mann-Whitney *U* tests; *P*-values are shown in each panel.

We next examined overall community composition at higher taxonomic levels. At the family level, responders exhibited increased relative abundance of Lachnospiraceae and Ruminococcaceae, whereas non-responders were enriched for Bacteroidaceae and Enterobacteriaceae, indicating a broad shift in community structure rather than isolated differences in individual taxa [Figure 2A]. 

To identify taxa that most strongly discriminated responders from non-responders, we applied LEfSe analysis, which highlighted multiple responder-enriched commensal Firmicutes at the species level, including *A. hadrus, R. intestinalis, *and* Fusicatenibacter saccharivorans, *whereas* Escherichia coli* and several other taxa were enriched in non-responders [Figure 2B]. These discriminative taxa were consistent with patterns observed in the abundance- and prevalence-based screening analyses, supporting their prioritization as response-associated candidates.

Consistent with the LEfSe results, non-parametric comparisons of species-level relative abundance demonstrated that *A. hadrus *[Figure 2C]*, R. intestinalis *[Figure 2D]*, *and* Fusicatenibacter saccharivorans *[Figure 2E] were significantly enriched in responders, whereas *Escherichia coli* was significantly enriched in non-responders [Figure 2F]. Relative abundance comparisons at the genus and species levels for additional candidate taxa showed concordant directional trends but did not consistently reach formal statistical significance, reflecting cohort heterogeneity and limited statistical power typical of clinical microbiome studies. These supporting analyses are therefore provided in the Supplementary Figure 1.

Together, these results indicate that responder microbiomes are characterized by enrichment of specific commensal Firmicutes taxa, while non-responder microbiomes show increased representation of potential pathobionts such as *Escherichia coli*. Importantly, candidate taxa were prioritized using an integrated framework that considered directionality of enrichment, prevalence across responders, consistency across analytical approaches, and suitability for downstream experimental validation, rather than reliance on statistical significance alone.

**Figure 3:**
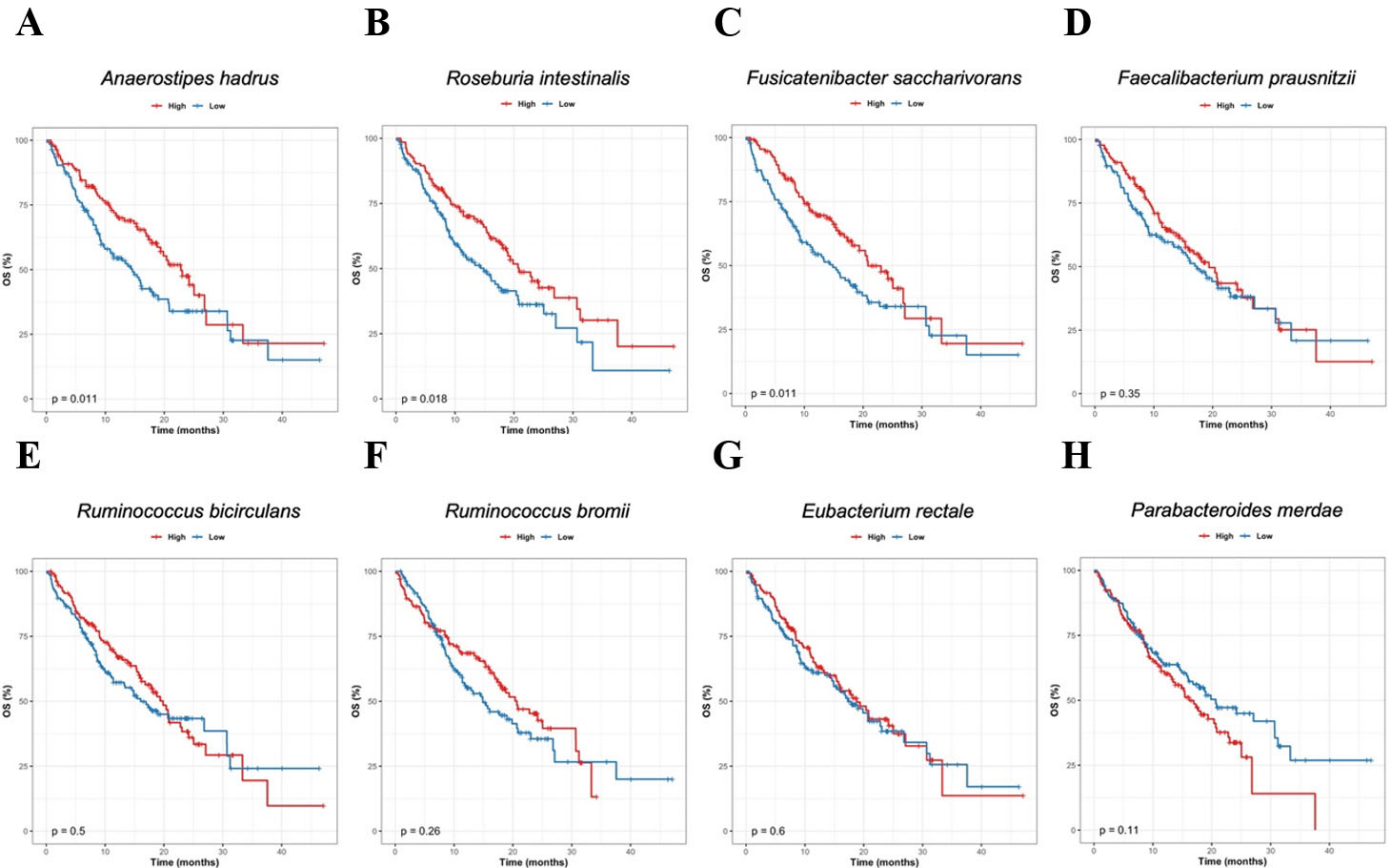
Association between responder-enriched species and overall survival. Kaplan-Meier OS curves for patients stratified by baseline relative abundance of responder-enriched candidate species. For each species, patients were divided into high- and low-abundance groups using the median baseline relative abundance as the cutoff, and OS was compared using the log-rank test. (A) *Anaerostipes hadrus*; (B) *Roseburia intestinalis*; (C) *Fusicatenibacter saccharivorans*; (D) *Faecalibacterium prausnitzii*; (E) *Ruminococcus bicirculans*; (F) *Ruminococcus bromii*; (G) *Eubacterium rectale*; (H) *Parabacteroides merdae. P*-values from log-rank tests are indicated in each panel. OS: Overall survival.

To assess the potential clinical relevance of the responder-associated taxa identified above, we next examined their relationship with OS. For species prioritized based on responder enrichment and consistency across the preceding analyses, patients were stratified into high- and low-abundance groups according to the median baseline relative abundance of each species, and OS was compared using Kaplan-Meier analysis with log-rank testing [Figure 3].

High baseline abundance of *A. hadrus* [Figure 3A], *R. intestinalis* [Figure 3B, and *Fusicatenibacter saccharivorans* [Figure 3C] was significantly associated with prolonged OS (*P* = 0.011, 0.018, and 0.014, respectively). For *F. prausnitzii* [Figure 3D], *Ruminococcus bicirculans* [Figure 3E], *Ruminococcus bromii *[Figure 3F] and* E. rectale* [Figure 3G], patients in the high-abundance groups showed a tendency toward improved OS compared with those in the low-abundance groups, although these differences did not reach statistical significance and were interpreted as suggestive trends. In contrast, higher baseline abundance of *Parabacteroides merdae* [Figure 3H] was associated with a trend toward poorer OS.

Exploratory Cox proportional hazards analyses adjusting for age, sex, or ECOG performance status yielded hazard ratios with similar direction and magnitude to the unadjusted analyses, indicating robustness to available clinical covariates.

Overall, these exploratory survival analyses indicate that several taxa enriched in responders are not only associated with radiologic response but may also be linked to improved survival outcomes, whereas certain non-responder-associated species may be associated with less favorable prognosis.

### Selection and characterization of the UJ-04 consortium

**Figure 4:**
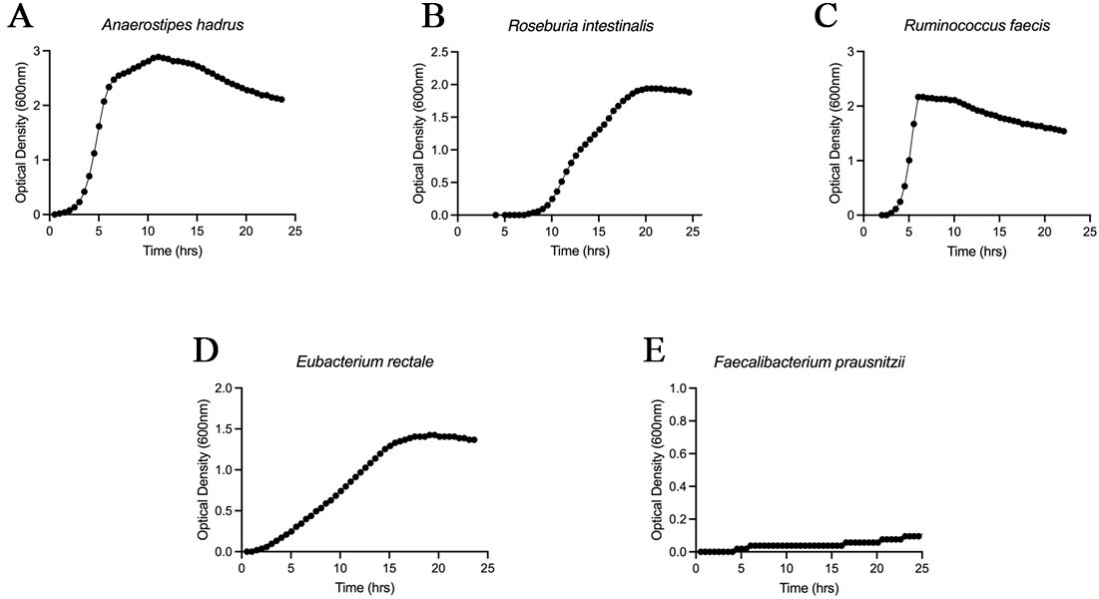
Growth characteristics of responder-enriched candidate species under anaerobic conditions. Growth curves of five responder-enriched candidate species: *Anaerostipes hadrus* (A); *Roseburia intestinalis* (B); *Ruminococcus faecis* (C); *Eubacterium rectale* (D); and *Faecalibacterium prausnitzii* (E). Cultures were incubated under anaerobic conditions at 37 °C, and OD_600_ was measured every 30 min for 24 h. Representative growth curves are shown. OD_600_: optical density at 600 nm.

Because subsequent *in vitro* and *in vivo* experiments required strains that could be reliably propagated, we evaluated the growth characteristics of responder-enriched candidate species. Among the taxa prioritized by the comparative metagenomic and survival analyses, we selected five species for which anaerobic isolates suitable for experimental use were available through public culture collections or in-house resources: *A. hadrus* [Figure 4A], *R. intestinalis* [Figure 4B], *R. faecis* [Figure 4C], *E. rectale* [Figure 4D], and *F. prausnitzii* [Figure 4E]. Seed cultures were pre-activated and inoculated at 2% (v/v) into pre-reduced GAM broth, and OD_600_ was monitored every 30 minutes for 24 h under strictly anaerobic conditions. Under these conditions, *A. hadrus, R. intestinalis, R. faecis,* and *E. rectale* exhibited robust growth and readily reached high optical densities, whereas *F. prausnitzii* showed very poor growth. Based on these growth characteristics, we selected the four robustly cultivable strains - *A. hadrus, R. intestinalis, R. faecis,* and *E. rectale* - and designated this four-strain consortium as UJ-04 for subsequent functional studies.

### Combination treatment with UJ-04 is associated with enhanced anti-PD-1 responses *in vivo*

**Figure 5:**
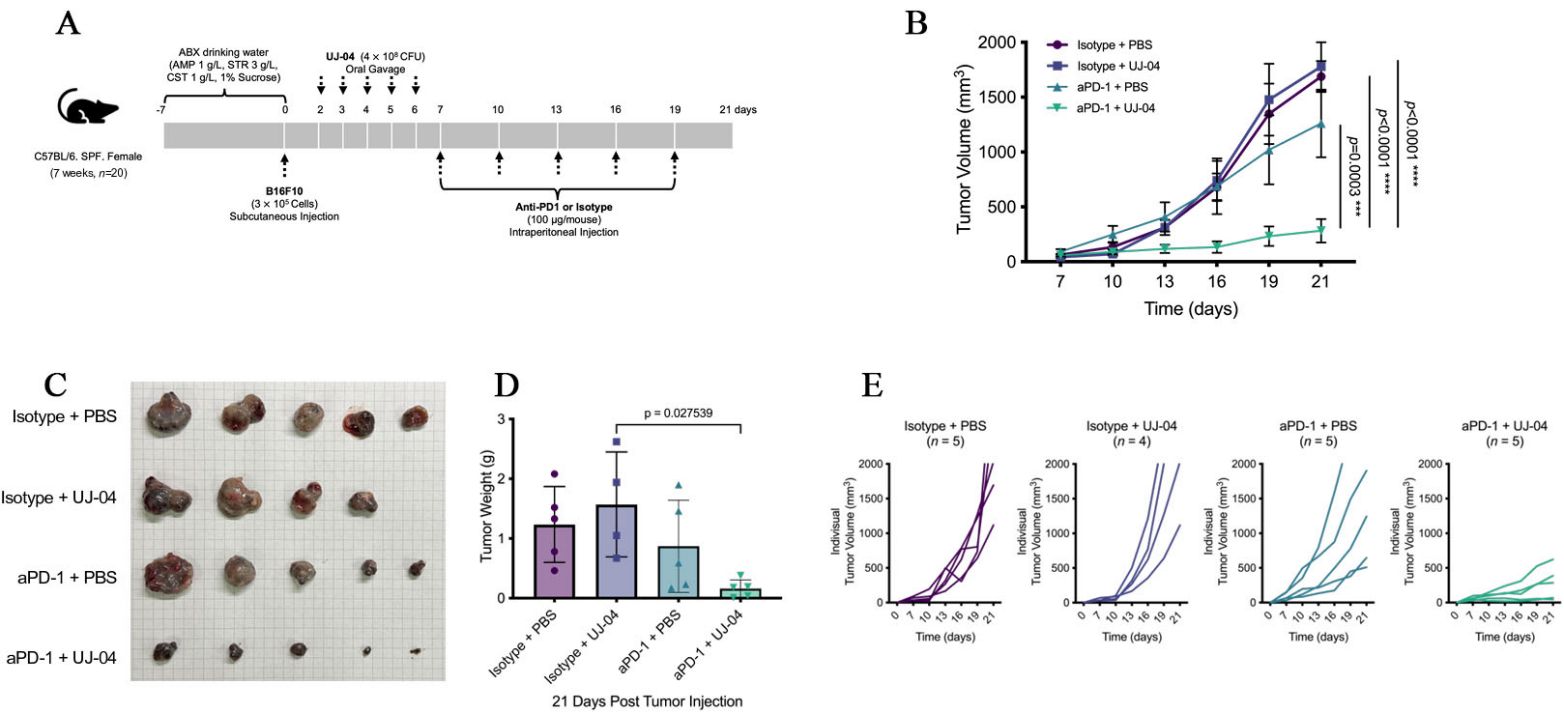
UJ-04 enhances the efficacy of anti-PD-1 in a mouse melanoma model. (A) Experimental schema for antibiotic treatment, tumor implantation, UJ-04 administration, and anti-PD-1 therapy. (B) Tumor growth curves for each treatment group. Data are shown as mean ± SEM (n = 4-5 mice per group). (C) Representative images of tumors resected at the experimental endpoint. (D) Endpoint tumor weight. (E) Individual tumor growth trajectories (“spaghetti” plots). Statistical analyses were performed as described in Methods. *, *P *< 0.05; **, *P *< 0.01; ***, *P *< 0.001; ****, *P *< 0.0001. ABX: Antibiotics; AMP: ampicillin; STR: streptomycin; CST: colistin; UJ-04: four-strain consortium; PBS: phosphate-buffered saline; SEM: standard error of the mean.

To assess whether UJ-04 could potentiate the efficacy of immune checkpoint blockade, we evaluated its effect in combination with anti-PD-1 in a subcutaneous B16-F10 melanoma model after antibiotic-mediated gut decontamination. Mice received an oral antibiotic cocktail (ampicillin 1 g/L, streptomycin 3 g/L, colistin 1 g/L, 1% w/v sucrose) in the drinking water from Day -7 to Day 0, followed by subcutaneous implantation of B16-F10 cells (3 × 10^5^ cells/mouse) on Day 0. UJ-04 (4 × 10^8^ CFU/mouse) or PBS was administered by oral gavage once daily on Days 2-6, and anti-PD-1 or isotype control antibody (100 μg/mouse, intraperitoneal) was given every 3 days from Day 7 to Day 19 [Figure 5A], yielding four treatment arms (isotype + PBS, isotype + UJ-04, anti-PD-1 + PBS, anti-PD-1 + UJ-04). Humane endpoints for tumor size and animal welfare were applied uniformly to all tumor-bearing mice as described in Methods.

To confirm effective microbiota depletion by antibiotics and subsequent community-level reconstitution following UJ-04 administration, longitudinal 16S ribosomal RNAs (rRNA) gene sequencing of pooled fecal samples was performed, revealing treatment-dependent shifts in microbial diversity and composition [Supplementary Figure 2A and B].

As expected, anti-PD-1 monotherapy reduced tumor growth compared with isotype control. Notably, the combination of anti-PD-1 with UJ-04 further suppressed tumor progression throughout the observation period [Figure 5B and C]. Two-way ANOVA (treatment × time) showed a significant treatment effect and interaction, and post hoc comparisons indicated that tumor volumes in the anti-PD-1 + UJ-04 group were significantly lower than those in the anti-PD-1 + PBS group at later time points, whereas UJ-04 alone did not differ from isotype + PBS. Consistent with the longitudinal measurements, endpoint tumor weight on Day 21 was lowest in the combination group and significantly reduced compared with anti-PD-1 monotherapy [Figure 5D], while UJ-04 alone again did not significantly alter tumor burden [Figure 5B and Supplementary Figure 3].

Importantly, inspection of individual tumor trajectories (“spaghetti” plots) revealed substantial inter-individual heterogeneity within treatment groups [Figure 5E]. No mice exhibited complete tumor regression in any group; however, several mice receiving the anti-PD-1 + UJ-04 combination displayed pronounced and sustained tumor growth delay, consistent with durable disease control over the observation period. In contrast, responses to anti-PD-1 monotherapy were more variable, with some mice showing partial growth delay but others exhibiting progressive tumor growth comparable to control-treated animals.

To confirm reproducibility, we performed an independent experiment using the same treatment schedule [Supplementary Figure 4A]. In this replicate cohort, the rank order of tumor growth was again isotype ≥ isotype + UJ-04 > anti-PD-1 + PBS > anti-PD-1 + UJ-04, and the combination group showed the greatest inhibition of tumor growth [Supplementary Figure 4B]. Although some pairwise comparisons between anti-PD-1 monotherapy and the combination did not reach statistical significance, the overall pattern closely mirrored the first experiment. Kaplan-Meier analysis of survival (with humane endpoints as above) likewise showed improved survival in anti-PD-1-treated groups compared with isotype controls, with the anti-PD-1 + UJ-04 group tending to survive the longest, despite the difference versus anti-PD-1 monotherapy not reaching statistical significance [Supplementary Figure 4C]. Together with the monotherapy data, these experiments indicate that UJ-04 does not exert direct antitumor activity on its own but is associated with greater tumor growth inhibition when combined with anti-PD-1 treatment in this model.

### Combination therapy is associated with increased intratumoral cytotoxic lymphocytes

**Figure 6:**
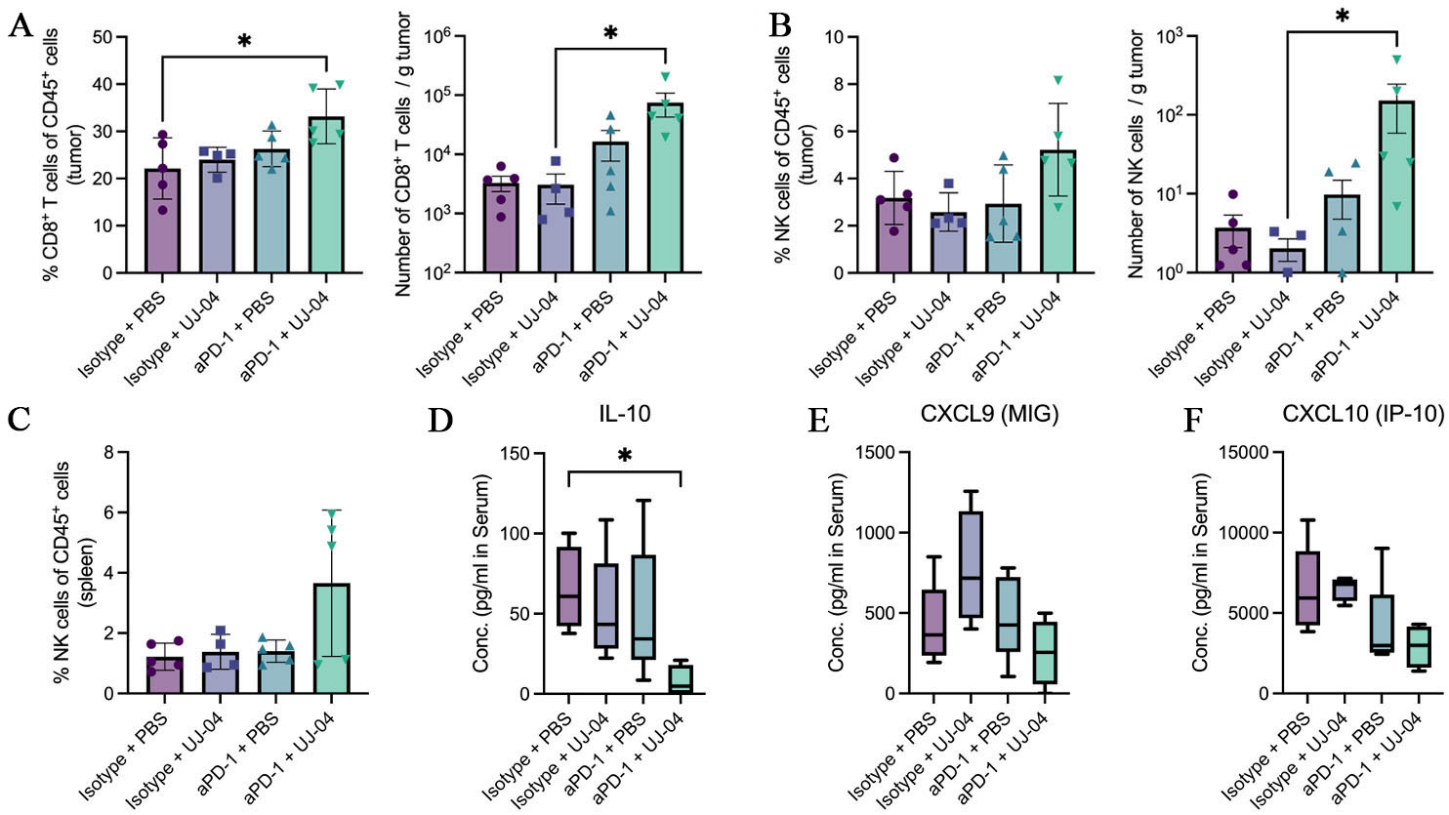
Immune profiling at the experimental endpoint. (A) Frequency of CD8^+^ T cells among CD45^+^ tumor cells, and absolute number of CD8^+^ T cells per gram of tumor; (B) Frequency of NK cells among CD45^+^ tumor cells, and absolute number of NK cells per gram of tumor; (C) Frequency of NK cells among CD45^+^ splenic cells; (D) Serum IL-10 concentrations measured by multiplex bead-based assay and presented as median with min-to-max whiskers (pg/mL); (E) Serum CXCL9 concentrations measured by multiplex bead-based assay and presented as median with min-to-max whiskers (pg/mL); (F) Serum CXCL10 concentrations measured by multiplex bead-based assay and presented as median with min-to-max whiskers (pg/mL). Statistical comparisons were performed using one-way ANOVA with Tukey’s multiple-comparison test or, where appropriate, the Kruskal-Wallis test with Dunn’s correction. Exact P values and significance annotations are indicated in the panels. *, *P *< 0.05. PBS: Phosphate-buffered saline; UJ-04: four-strain consortium; aPD-1: anti-programmed cell death protein 1 antibody; IL-10: interleukin 10; MIG: monokine induced by gamma interferon; IP-10: interferon gamma-induced protein 10; NK: natural killer; CXCL: C-X-C motif chemokine ligand; ANOVA: analysis of variance.

At the experimental endpoint (Day 21), tumors and spleens were harvested for immune profiling by flow cytometry, and serum was collected for cytokine analysis. Gating strategies for tumor-infiltrating and splenic lymphocytes are shown in Figure 6A-C, respectively. In tumors, leukocytes were identified through sequential gating on lymphocytes, live cells, singlets, and CD45^+^ events, followed by delineation of CD8^+^ T cells and NK cells [Supplementary Figure 5A]. In spleens, NK cells were defined within the CD45^+^CD3^-^ compartment [Supplementary Figure 5B].

In the tumor, CD8^+^ T-cell infiltration was evaluated using two representations of the same cell population. The frequency of CD8^+^ T cells among CD45^+^ cells was significantly increased in the anti-PD-1 plus UJ-04 group compared with the isotype plus PBS control. When the same CD8^+^ T-cell population was expressed as absolute numbers normalized to tumor weight, the number of CD8^+^ T cells per gram of tumor was also higher in the combination group compared with isotype plus UJ-04 alone [Figure 6A].

Tumor-infiltrating NK cells were analyzed in the same manner. The percentage of NK cells among CD45^+^ tumor cells did not differ significantly between groups, although the anti-PD-1 plus UJ-04 group tended to show higher values. However, NK cells per gram of tumor were significantly increased in the combination group compared with isotype plus UJ-04 [Figure 6B]. In the spleen, NK cell frequencies showed no statistically significant differences between groups, but the combination group displayed the highest values [Figure 6C].

To assess whether these cellular changes were accompanied by alterations in systemic immune mediators, serum cytokines were measured using a multiplex bead-based assay and are presented as median concentrations with min-to-max whiskers. IL-10 levels were markedly reduced in the anti-PD-1 plus UJ-04 group compared with the isotype plus PBS control, reaching statistical significance [Figure 6D]. In contrast, CXCL9 and CXCL10 levels did not differ significantly between groups but showed a consistent decreasing pattern across treatments, with the lowest concentrations observed in the combination group [Figure 6E and F].

Overall, combination therapy was associated with increased intratumoral CD8^+^ T cells and NK cells and reduced circulating IL-10 at the endpoint. These immune changes were observed at the experimental endpoint and are reported as associated phenotypes rather than definitive mechanistic determinants.

## DISCUSSION

Clinical and preclinical studies have linked gut microbiome composition to outcomes of immune checkpoint blockade^[23,27-30,34,35]^. Our results add functional support to this body of work by demonstrating that a defined bacterial consortium selected from responder-biased taxa can enhance anti-PD-1 efficacy *in vivo* and is accompanied by immune features aligned with therapeutic response. In line with its proposed role as an immunomodulatory adjunct, UJ-04 primarily potentiated the efficacy of PD-1 blockade rather than acting as a stand-alone antitumor agent.

Importantly, the present findings should not be interpreted as evidence of tumor-type-specific therapeutic efficacy or as a melanoma-directed treatment strategy. Instead, the *in vivo* experiments were designed to address a broader immunological question: whether a responder-informed microbial consortium can function as an immunomodulatory adjunct that enhances PD-1 blockade by shaping host antitumor immunity.

We re-analyzed publicly available metagenomic datasets from patients treated with anti-PD-1 to nominate responder-associated taxa and subsequently assembled a defined, cultivable consortium (UJ-04) for functional testing. The strains selected for UJ-04 comprise commensal anaerobes that have been recurrently linked to favorable immune checkpoint outcomes across multiple cohorts^[27,36]^, and related taxa have been implicated in promoting antitumor immunity in preclinical studies^[37]^. Several UJ-04 members belong to Clostridiales/Lachnospiraceae-Ruminococcaceae lineages that are frequently enriched in responders across independent studies of PD-1 blockade, supporting their prioritization as candidates for functional evaluation^[38]^. Importantly, responder-enriched taxa are known to vary across cohorts depending on clinical context, geography, and analytical approaches; accordingly, UJ-04 was assembled to represent a functionally coherent set of responder-associated microbes rather than the most statistically significant taxa in isolation^[27,34,36,39]^. We acknowledge that SD represents a clinically heterogeneous category under PD-1 blockade and that RECIST-based response definitions may incompletely capture durable clinical benefit. Importantly, the robustness of the responder-associated microbiome signatures to alternative, more stringent response definitions supports the interpretation that these associations are not driven by classification artifacts related to SD.

Although *E. rectale* did not exhibit the strongest individual statistical signal in all analyses, its inclusion in the UJ-04 consortium reflects the multi-dimensional selection logic underlying consortium assembly. In our analyses, *E. rectale* consistently showed responder-associated directionality and high prevalence within the responder cohort, despite more modest effect sizes in abundance and survival stratification. In addition, *E. rectale* demonstrated robust and reproducible anaerobic growth, enabling reliable preparation and dosing for *in vivo* experiments. Together, these features supported its inclusion as part of a functionally coherent and experimentally tractable consortium, rather than selection based solely on single-metric statistical dominance.

Mechanistically, prior studies have implicated microbiota-derived short-chain fatty acids (SCFAs), including butyrate and propionate, in supporting cytotoxic T-lymphocyte programs through metabolic and epigenetic reprogramming, including enhanced IFN-γ and granzyme B expression^[40,41]^. Although SCFAs or other microbial metabolites were not measured in the present study, these findings provide a plausible biological context for how responder-associated commensals may influence effector T-cell function during PD-1 blockade, without implying a direct causal mechanism in this model. Notably, the functional convergence of UJ-04 members toward SCFA-producing taxa was observed retrospectively and was not used as an a priori selection criterion.

In the B16-F10 melanoma model, UJ-04 alone showed limited antitumor activity, whereas combination treatment with anti-PD-1 reduced tumor growth more effectively than anti-PD-1 monotherapy. Endpoint immune profiling revealed increased intratumoral CD8^+^ T-cell and NK-cell metrics in the combination group, with similar trends observed in peripheral compartments. Together, these observations indicate that UJ-04 functions in a context-dependent manner, modulating the immune milieu to enhance checkpoint efficacy rather than exerting direct cytotoxic effects. This distinction is consistent with clinical observations that microbiome primarily shapes immunotherapy responsiveness rather than independently driving tumor regression. In this context, the B16-F10 melanoma model - characterized by limited responsiveness to PD-1 monotherapy and a highly immunosuppressive TME - served as a stringent system to evaluate immune-adjuvant potential rather than cancer-type-specific benefit. This is consistent with prior studies demonstrating that B16-F10 melanoma represents an immunologically cold and PD-1-refractory syngeneic model with low baseline CD8^+^ T-cell infiltration, in which sensitization to PD-1 blockade requires modulation of tumor-intrinsic or immune-regulatory pathways^[42]^.

In parallel, longitudinal 16S rRNA gene sequencing of pooled fecal samples was performed to confirm effective antibiotic-mediated microbiota depletion and subsequent community-level reconstitution following microbial intervention [Supplementary Figure 2]. Antibiotic treatment induced a pronounced reduction in fecal microbial diversity, followed by partial recovery after UJ-04 or PBS administration. Notably, higher alpha diversity was observed at later time points in the aPD-1 + UJ-04 group than in PBS-treated controls. These findings provide descriptive support that UJ-04 administration was associated with distinct temporal shifts in the gut microbial ecosystem, without implying stable intestinal colonization or direct causal linkage to antitumor efficacy.

At the experimental endpoint, combination therapy was associated with increased intratumoral CD8^+^ T cells and NK cells together with reduced circulating IL-10, a cytokine widely implicated in systemic immune suppression. In poorly immunogenic melanoma models such as B16, regulatory immune circuits - including regulatory T cell-mediated suppression - have been shown to actively prevent the development of effective CD8^+^ T cell-dependent antitumor immunity, highlighting the functional relevance of relieving systemic immunosuppressive signals in this context^[43]^. Although soluble mediators were not assessed longitudinally, this cellular and cytokine profile is consistent with an immune context favorable for PD-1 responsiveness, characterized by enhanced representation of cytotoxic lymphocytes rather than generalized inflammatory activation. The absence of antitumor efficacy with UJ-04 monotherapy further supports the interpretation that the consortium modulates host immune tone to potentiate checkpoint blockade.

An additional observation was the reduction of circulating CXCL9 and CXCL10 in the combination group at the endpoint. CXCL9 and CXCL10 are IFN-γ-inducible C-X-C motif chemokine receptor 3 (CXCR3) ligands that play central roles in effector lymphocyte trafficking and positioning within inflamed tissues^[44]^, and IFN-γ-associated chemokine programs have been repeatedly linked to clinical benefit from PD-1 blockade^[45-47]^. While reduced serum levels might initially appear counterintuitive, a biologically plausible interpretation is that effective recruitment of effector lymphocytes into the TME may be accompanied by preferential local retention or consumption of these chemokines within tumor tissues, resulting in lower circulating concentrations at later stages of treatment. In this context, decreased serum CXCL9 and CXCL10 could reflect successful spatial redistribution of chemokine signaling toward the tumor rather than impaired IFN-γ-driven immune activation. Consistent with this interpretation, the combination group exhibited increased intratumoral CD8^+^ T-cell and NK-cell metrics at the endpoint. However, because chemokine levels were not directly assessed within tumor tissues, this interpretation remains speculative and warrants future validation using paired serum and intratumoral measurements.

Several defined microbial consortia have previously been reported to enhance antitumor immunity or improve the efficacy of immune checkpoint blockade, including Clostridiales-based consortia and Bifidobacterium-enriched communities, as well as responder-derived fecal microbiota transplantation^[24,25,34,48]^. Compared with these approaches, UJ-04 represents a streamlined, responder-informed consortium composed of four cultivable commensal anaerobes selected based on consistent enrichment in anti-PD-1 responders across metagenomic and survival analyses. Unlike larger consortia designed to broadly stimulate antitumor immunity, UJ-04 was evaluated specifically as an adjuvant to PD-1 blockade, rather than as a stand-alone antitumor intervention. Despite differences in taxonomic composition across studies, UJ-04 and previously reported consortia converge on a shared functional theme, namely enrichment of obligate anaerobic commensals recurrently associated with favorable immunotherapy responses^[28,35]^.

Mechanistic explanations for such consortium effects are likely multifactorial in nature. From a mechanistic perspective, the interaction between the gut microbiome and antitumor immunity is increasingly understood as a multi-level, function-centered process, rather than a phenomenon that relies solely on stable intestinal colonization by specific bacterial strains. Recent conceptual frameworks suggest that the microbiome can modulate the efficacy of immune checkpoint blockade through functional outputs, such as microbe-derived metabolites and immune-regulatory signals, even in the absence of durable engraftment^[49]^. In line with this view, postbiotic-mediated sensitization of tumors to PD-1 blockade has been reported, demonstrating that microbial products alone can enhance antitumor immune responses independently of persistent live bacterial presence^[50]^. Taken together, these observations provide a conceptual framework in which the effects of UJ-04 may be interpreted as functional modulation of the host antitumor immune environment, despite the absence of direct evidence for long-term consortium colonization or a fully resolved molecular mechanism in the present study.

Several limitations should be acknowledged. First, longitudinal fecal microbiota profiling was performed using pooled samples, precluding assessment of inter-individual variability and formal statistical testing of microbiota changes. Accordingly, the 16S rRNA gene sequencing data should be interpreted as descriptive and exploratory, providing community-level context rather than definitive evidence of stable colonization or causal linkage to antitumor efficacy. In addition, because responder-associated taxa were nominated from an NSCLC cohort while *in vivo* validation was performed in a melanoma model, the present findings should be interpreted as evidence of generalizable immunomodulatory potential rather than tumor-type-specific efficacy. This study relied on a single clinical cohort for metagenomic nomination, and responder-associated taxa are known to vary across cohorts. A key limitation of the survival analyses is the inability to adjust for several important clinical determinants of outcome in advanced NSCLC, such as smoking history, tumor histology, disease burden, and treatment line or regimen. Although these variables were described in the original cohort report, they were not available at the individual-patient level in the publicly accessible dataset. Consequently, the survival analyses should be interpreted as exploratory and hypothesis-generating, rather than as definitive evidence of independent microbiome effects. While our selection strategy emphasized convergence across analytical metrics and prior literature, broader multi-cohort validation would strengthen generalizability. In addition, UJ-04 colonization and persistence were not directly verified by sequencing-based approaches, leaving engraftment as an assumption. The *in vivo *experiments were conducted in a single tumor model with modest sample size, and causal testing of specific immune pathways - such as CXCR3-mediated trafficking or CD8^+^ and NK-cell dependence - was not performed.

Future studies should therefore incorporate longitudinal profiling of serum and intratumoral cytokines and chemokines, direct assessment of consortium colonization dynamics, and mechanistic perturbations such as selective immune cell depletion or chemokine receptor blockade. Extending validation to additional tumor models and cancer types, complemented by spatial immune analyses, will further clarify the translational potential of defined microbial consortia.

In summary, this study demonstrates that a responder-informed, cultivable gut microbial consortium can potentiate PD-1 blockade *in vivo* through modulation of host antitumor immunity. By directly linking clinical metagenomic re-analysis with functional validation and immune profiling, these findings support microbiome-based adjunct strategies for immunotherapy and provide a conceptual foundation for future mechanistic and translational investigations.

## DECLARATIONS

### Availability of data and materials

The clinical metagenomic datasets analyzed in this study were originally generated and published by previous studies and are publicly available in the National Center for Biotechnology Information (NCBI) Sequence Read Archive (SRA) under BioProject accession PRJNA751792. Species-level taxonomic abundance tables and corresponding clinical metadata were obtained from the publicly available supplementary materials of the original publication and were re-analyzed in this study.

Custom scripts used for data processing, statistical analysis, and figure generation, together with processed data tables generated in this study, will be deposited in a publicly accessible GitHub repository entitled “MRR-2025-117_microbiome_analysis” and made openly available upon acceptance of the manuscript.

### Copyright

© The Author(s) 2026.
