# Supplementary material for: A responder-informed gut microbial consortium enhances anti-PD-1 efficacy in a mouse cancer model
Source: Microbiome Res Rep. 2026 Feb 9;5(1):2. doi: 10.20517/mrr.2025.117 (PMC13091084; doi:10.20517/mrr.2025.117)
Supplement: Supplementary file 1 [file mrr-5-1-2-SupplementaryMaterials.pdf]

DOI: 10.20517/mrr.xxxx.xx

Microbiome Research Reports

Supplementary Materials

A responder-informed gut microbial consortium enhances anti-PD-1 efficacy in a mouse cancer model

Uk Jin Jeong[1,2], Mohammed Ali[3], Yun Jee Park[1,2], Jin Sun You[1,4], Sang Sun Yoon[1,2,4,5,6]

[1]Department of Microbiology and Immunology, Yonsei University College of Medicine, Seoul 03722, Republic of Korea.
[2]Brain Korea 21 Project for Medical Sciences, Yonsei University College of Medicine, Seoul 03722, Republic of Korea.
[3]Section of Hematology, Yale Cancer Center, Yale University School of Medicine, New Haven, CT 06511, USA.
[4]Institute for Immunology and Immunological Diseases, Yonsei University College of Medicine, Seoul 03722, Republic of Korea.
[5]Severance Biomedical Science Institute, Yonsei University College of Medicine, Seoul 03722, Republic of Korea.
[6]BioMe Inc., Seoul 02455, Republic of Korea.

Correspondence to: Prof. Sang Sun Yoon, Dr. Jin Sun You, Department of Microbiology and Immunology, Yonsei University College of Medicine, Seoul 03722, Republic of Korea. E-mail: sangsun_yoon@yuhs.ac; jinsunyou@yuhs.ac

© The Author(s) 2021. Open Access This article is licensed under a Creative Commons Attribution 4.0 International License (https://creativecommons.org/licenses/by/4.0/), which permits unrestricted use, sharing, adaptation, distribution and reproduction in any medium or format, for any purpose, even commercially, as long as you give appropriate credit to the original author(s) and the source, provide a link to the Creative Commons license, and indicate if changes were made.

ORCID: Sang Sun Yoon (0000-0003-2979-365X), Jin Sun You (0009-0006-9958-473X)

Supplementary Methods

DNA extraction and quantification

Fecal DNA extraction and quantification were performed by a commercial service provider (Macrogen, Seoul, Republic of Korea). Total microbial DNA was isolated from fecal samples using the DNeasy PowerSoil Pro Kit (Qiagen, Hilden, Germany) following the manufacturer's recommended protocol. DNA concentration was measured fluorometrically using PicoGreen reagents on a VICTOR Nivo™ multimode plate reader (PerkinElmer, Waltham, MA, USA).

16S rRNA gene library preparation and sequencing

The 16S ribosomal RNA (rRNA) gene library preparation and sequencing were conducted by Macrogen according to the Illumina 16S Metagenomic Sequencing Library Preparation guidelines. Sequencing libraries targeting the bacterial 16S rRNA gene were generated according to the Illumina 16S Metagenomic Sequencing Library Preparation guidelines. Briefly, 5 ng of input genomic DNA was amplified using a universal primer set targeting the V3–V4 hypervariable regions, containing Illumina adapter overhang sequences. Polymerase Chain Reaction (PCR) reactions were performed using a 5× reaction buffer, deoxynucleotide triphosphates (dNTPs), universal forward and reverse primers (500 nM each), and Herculase II Fusion DNA polymerase (Agilent Technologies, Santa Clara, CA, USA).

The initial PCR amplification was carried out with an initial denaturation at 95 °C for 3 min, followed by 25 cycles of denaturation at 95°C for 30 s, annealing at 55 °C for 30 s and extension at 72 °C for 30 s, with a final extension at 72 °C for 5 min. Amplicons from the first-round PCR were purified using AMPure XP beads (Beckman Coulter, Beverly, MA, USA).

Indexing and final library amplification were performed using the Nextera XT Index Kit, with PCR conditions identical to the first amplification except for a reduced cycle number (10 cycles). Indexed PCR products were purified again using AMPure XP beads. Final libraries were quantified using PicoGreen assays on the VICTOR Nivo™ system and assessed for fragment size distribution using the TapeStation D1000 ScreenTape system (Agilent Technologies, Waldbronn, Germany).

Libraries were normalized, pooled, and quantified by quantitative PCR using the KAPA Library Quantification Kit for Illumina sequencing platforms (Roche, Basel, Switzerland). Sequencing was performed on the Illumina MiSeq i100 platform (Illumina, San Diego, CA, USA) according to the manufacturer's instructions.

Supplementary Figures

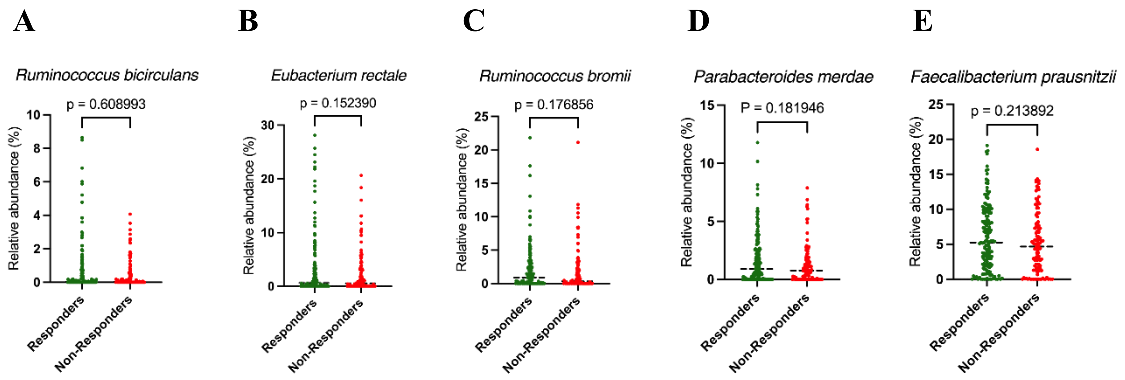

Supplementary Figure 1. Additional species-level relative abundance comparisons. (A-E) Relative abundance of selected species in responders and non-responders. Panels show *Ruminococcus bicirculans* (A), *Eubacterium rectale* (B), *Ruminococcus bromii* (C), *Parabacteroides merdae* (D), and *Faecalibacterium prausnitzii* (E). Relative abundances are expressed as percentages. Group comparisons were performed using two-sided Mann-Whitney *U* tests; p values are shown in each panel. Relative abundance comparisons were analyzed and visualized using GraphPad Prism (version 10.1.1).

Jeong *et al. Microbiome Res Rep* 2025;Volume:Number | http://dx.doi.org/10.20517/mrr.xxxx.xx

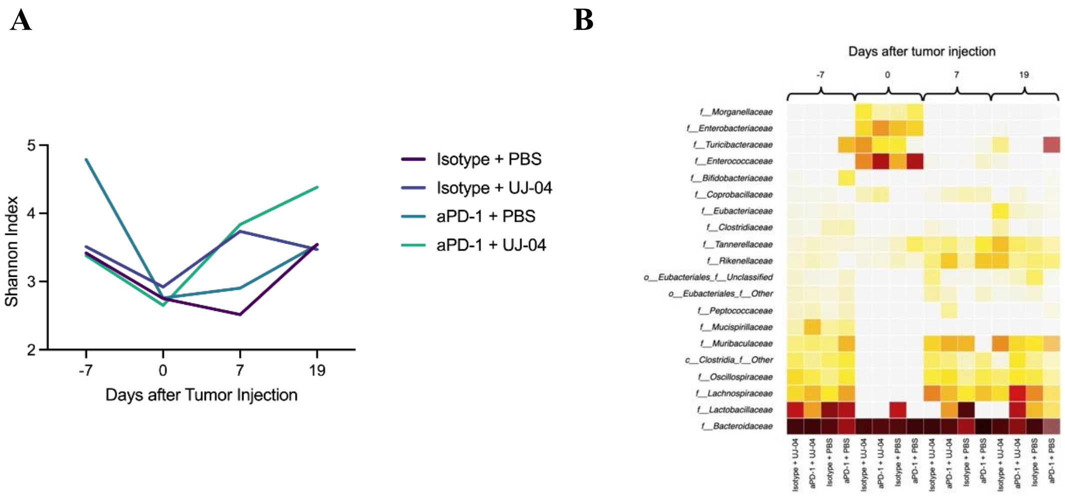

Supplementary Figure 2. Longitudinal changes in fecal microbiota diversity and composition during antibiotic depletion and UJ-04 treatment. (A) Fecal microbiota alpha diversity assessed by Shannon index at four longitudinal time points: before antibiotic treatment (day -7), after 7 days of antibiotic treatment (day 0), after five oral gavage administrations of UJ-04 consortium or PBS (day 7 post tumor implantation), and at a late time point (day 19 post tumor implantation). Alpha diversity values were visualized by the authors using GraphPad Prism (version 10.1.1). At each time point, fecal samples from five mice per group were pooled and subjected to 16S rRNA gene sequencing. (B) Heatmap of family-level fecal microbiota composition across treatment groups and time points, showing relative abundances of major bacterial families. The heatmap was generated by a commercial microbiome analysis service provider (Macrogen, Seoul, Republic of Korea) as part of the primary microbiome analysis pipeline and was subsequently reannotated by the authors to reflect experimental group assignments.

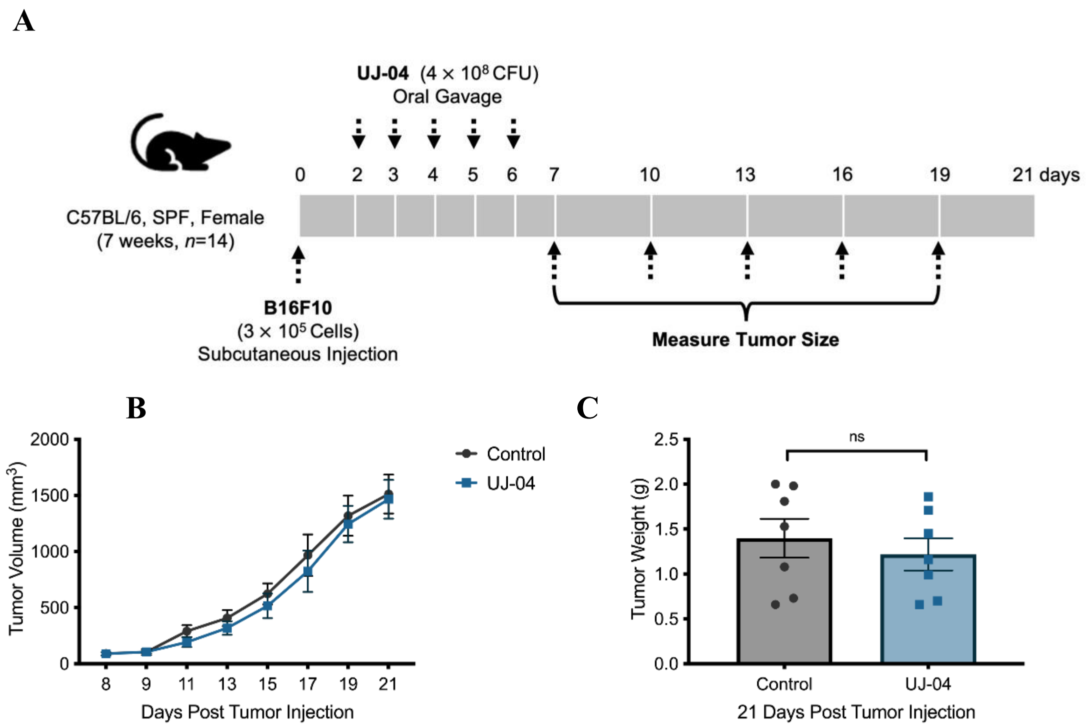

Supplementary Figure 3. *In vivo* efficacy of UJ-04 monotherapy. (A) Experimental scheme. C57BL/6 female mice (7 weeks old, n = 7 per group) were subcutaneously injected with B16F10 melanoma cells (3×10^5 cells/mouse, day 0). From day 2 after tumor implantation, mice received either PBS (Control) or the UJ-04 consortium (4×10^8 CFU/mouse) by oral gavage five times. Tumor size was measured at the indicated time points, and mice were sacrificed on day 21 for endpoint analysis. (B) Tumor growth curves. Tumor volume was monitored every 2-3 days up to day 21. Data are presented as mean ± SEM (n = 7 per group). (C) Tumor weight at endpoint. Tumors were excised and weighed on day 21. Data are shown as mean ± SEM (n = 7 per group). Tumor growth curves and endpoint tumor weight plots were generated using GraphPad Prism (version 10.1.1). Mouse icons were obtained from the built-in icon library of Microsoft PowerPoint.

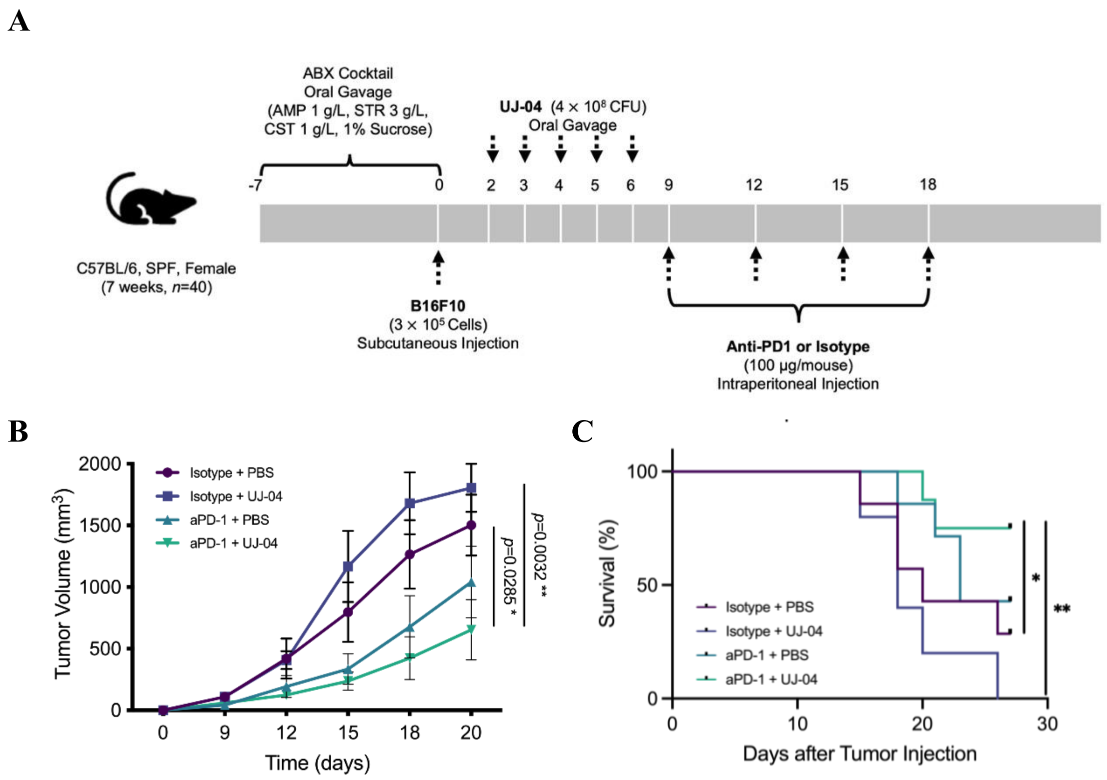

Supplementary Figure 4. Independent replicate *in vivo* experiment. (A) Experimental schema for the replicate study. (B) Tumor growth curves from an independent replicate experiment using the same treatment schedule, analyzed as in Figure 5. (C) Kaplan–Meier survival curves from the replicate experiment. Survival was monitored under the same humane endpoint criteria applied to all *in vivo* tumor experiments (see Methods). Survival differences between groups were analyzed using the log-rank (Mantel-Cox) test. *, $P < 0.05$; **, $P < 0.01$. Tumor growth curves and Kaplan-Meier survival analyses were performed and visualized using GraphPad Prism (version 10.1.1). Mouse icons were obtained from the built-in icon library of Microsoft PowerPoint.

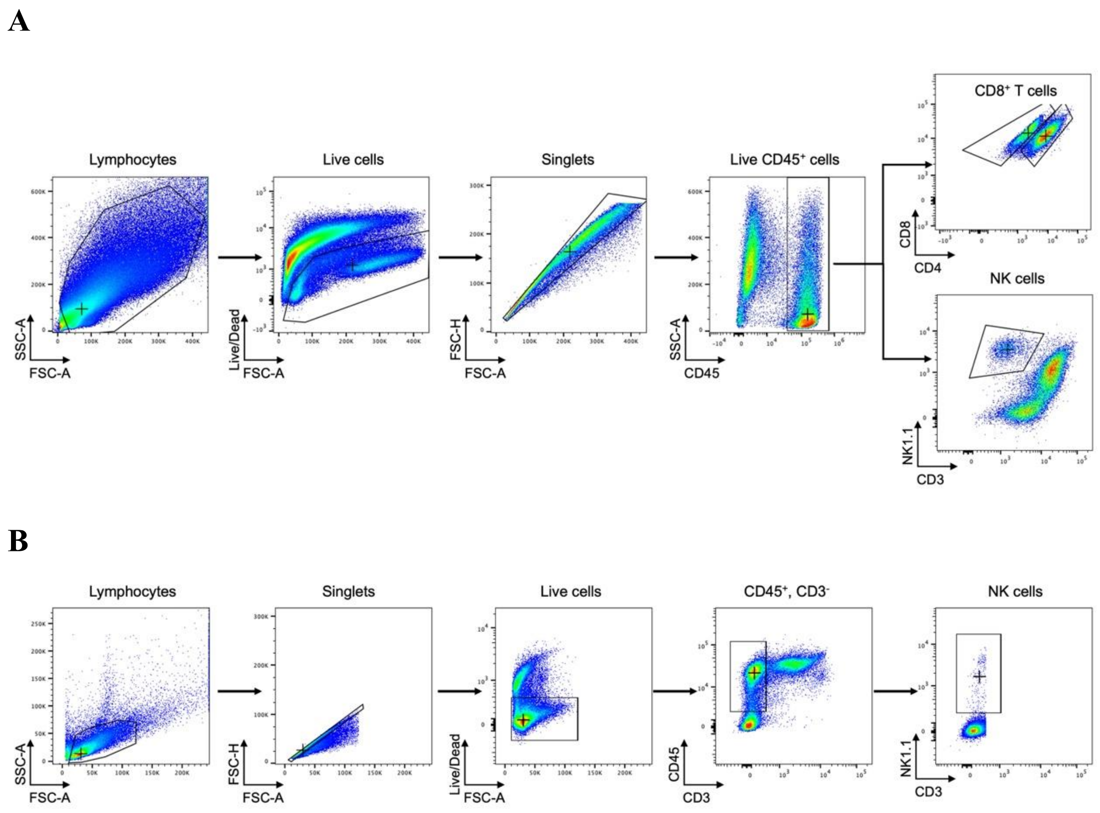

Supplementary Figure 5. Representative flow cytometry gating strategies. (A) Representative gating strategy for tumor-infiltrating lymphocytes: lymphocytes → live cells → singlets → $CD45^+$ cells → $CD8^+$ T cells and NK cells. (B) Representative gating strategy for splenic lymphocytes: lymphocytes → singlets → live cells → $CD45^+CD3^-$ cells → NK cells. Flow cytometry data were analyzed and gating strategies were visualized using FlowJo (version 10.10.0).

Supplementary Table 1. Baseline clinical characteristics of the anti–PD-1–treated patient cohort

Demographics	Responders		Non-Responders		Overall patients	
	N	%	N	%	N	%
	155	57.6	114	42.4	269	100
**Age**	66.0	-	64.0	-	65.0	-
**IQR**	60.0-72.0	-	59.0-70.8	-	59.0-71.0	-
**Sex**						
**Male**	107	69.0	72	63.2	179	66.5
**Female**	48	31.0	42	36.8	90	33.5
**Best overall response**						
**Complete response**	7	4.5	0	0.0	7	2.6
**Partial response**	63	40.6	0	0.0	63	23.4
**Stable disease**	85	54.8	0	0.0	85	31.6
**Progressive disease**	0	0.0	114	100.0	114	42.4
**ECOG**						
**0-1**	127	81.9	91	79.8	218	81.0
**≥2**	13	8.4	17	14.9	30	11.2
**unknown**	15	9.7	6	5.3	21	7.8

Demographic and clinical features of responders and non-responders included in the metagenomic re-analysis are summarized. Response categories were defined according to RECIST v1.1 criteria. Clinical metadata were obtained from the publicly available dataset reported in [Derosa et al., 2022], and responder/non-responder classification was newly assigned in this study based on the reported RECIST outcomes.

Supplementary Table 2. Characteristics of bacterial strains included in the UJ-04 consortium

Species	Strain source	Host origin	Culture conditions	Prior association with ICI response
Anaerostipes hadrus	YSflora®[33]	Human (gut commensal)	Strict anaerobic conditions; Gifu Anaerobic Medium; 37°C	Enriched in anti–PD-1 responders in clinical cohorts [36]
Roseburia intestinalis	Korean Collection for Type Cultures(KCTC)	Human (gut commensal)	Strict anaerobic conditions; Gifu Anaerobic Medium; 37°C	Associated with favorable immunotherapy outcomes [22], [36], [38]
Eubacterium rectale	Korean Collection for Type Cultures(KCTC)	Human (gut commensal)	Strict anaerobic conditions; Gifu Anaerobic Medium; 37°C	Recurrently enriched in responders across cohorts [23]
Ruminococcus faecies	YSflora®[33]	Human (gut commensal)	Strict anaerobic conditions; Gifu Anaerobic Medium; 37°C	Linked to immune modulation in prior studies

Strain source, host origin, culture conditions, and previously reported associations with immune checkpoint inhibitor (ICI) response are summarized for each consortium member.
